# Preliminary Study on the Combination Effect of Clindamycin and Low Dose Trimethoprim-Sulfamethoxazole on Severe *Pneumocystis* Pneumonia After Renal Transplantation

**DOI:** 10.3389/fmed.2022.827850

**Published:** 2022-05-06

**Authors:** Zhun-Yong Gu, Wen-Jun Liu, Dan-Lei Huang, Yu-Jing Liu, Hong-Yu He, Cheng Yang, Yi-Mei Liu, Ming Xu, Rui-Ming Rong, Du-Ming Zhu, Zhe Luo, Min-Jie Ju

**Affiliations:** ^1^Department of Critical Care Medicine, Zhongshan Hospital, Fudan University, Shanghai, China; ^2^Department of Nursing, Zhongshan Hospital, Fudan University, Shanghai, China; ^3^Department of Urology Surgery, Zhongshan Hospital, Fudan University, Shanghai, China

**Keywords:** clindamycin, trimethoprim-sulfamethoxazole, *Pneumocystis* pneumonia, renal transplantation, combination

## Abstract

**Objective:**

Evaluate the effect of the combination of clindamycin with low-dose trimethoprim-sulfamethoxazole (TMP/SMX) regimen on sever *Pneumocystis* pneumonia (PCP) after renal transplantation.

**Method:**

20 severe PCP patients after renal transplantation were included in this historical-control, retrospective study. A 10 patients were treated with the standard dose of TMP/SMX (T group), the other 10 patients were treated with the combination of clindamycin and low dose TMP/SMX (CT group).

**Results:**

Although there was no significant difference in the hospital survival between the two groups, the CT protocol improved the PaO2/FiO2 ratio more significantly and rapidly after the 6th ICU day (1.51 vs. 0.38, *P* = 0.014). CT protocol also ameliorated the pulmonary infiltration and the lactate dehydrogenase level more effectively. Moreover, the CT protocol reduced the incidence of pneumomediastinum (0 vs. 50%, *P* = 0.008), the length of hospital staying (26.5 vs. 39.0 days, *P* = 0.011) and ICU staying (12.5 vs. 22.5 days, *P* = 0.008). Furthermore, more thrombocytopenia (9/10 vs. 3/10, *P* = 0.020) was emerged in the T group than in the CT group. The total adverse reaction rate was much lower in the CT group than in the T group (8/80 vs. 27/80, *P* < 0.001). Consequently, the dosage of TMP/SMX was reduced in 8 patients, while only 2 patients in the CT group received TMP/SMX decrement (*P* = 0.023).

**Conclusion:**

The current study proposed that clindamycin combined with low-dose TMP/SMX was more effective and safer the than single use of TMP/SMX for severe PCP patients after renal transplantation (NCT 04328688).

## Introduction

*Pneumocystis* pneumonia (PCP) is a severe disease with high morbidity and mortality, which almost exclusively affects immunocompromised patients (1–4), including solid organ transplant (SOT). SOT was one of the most frequent underlying diseases among non-HIV-PCP patients (3). For SOT patients, the overall incidence of PCP varies from 5 to 15% (5, 6), which increases along with the increasing numbers of transplantations. The incidence is also influenced by the type of the transplanted organ and the immunosuppressive regimen (7). Non-HIV-PCP will progress more rapidly than HIV-PCP, predominantly in hypoxemia (5, 8, 9). Consequently, studies have proposed that the mortality of non-HIV-PCP is as high as 30 – 60% (3, 10, 11), which is significantly higher than that of HIV-PCP (5). How to effectively treat severe PCP after SOT has become an urgent problem to be solved.

At present, trimethoprim-sulfamethoxazole (TMP/SMX) is still recommended as the first-line treatment for PCP after SOT (12, 13), and the standard dose is 15–20 mg/kg/d TMP combined with 75–100 mg/kg/d SMX. However, these dosages are resulted from some small, observational studies during 1970s and 1980s (14–16), without randomized control. Hence, the optimal dose of TMP/SMX for PCP after SOT remains ambiguous. Furthermore, the standard dosages are more likely to cause side effects (bone marrow depression, hyperkalemia and nephrotoxicity, et al.) due to the large dose and poor compliance with medication (17). To reduce the adverse reactions of TMP/SMX and improve the adherence to medication, the prevention strategies of PCP after SOT were referred, including the escalating protocol, the half dose protocol and the single tablet chemoprophylaxis protocol (18–20). As a result, the medium dose (10 mg/kg/d TMP) (21), decreasing dose (22, 23) and low dose strategy (4–10 mg/kg/d TMP) (17) are used to treat SOT-PCP. However, the effect after dose modification remains controversial (14, 24, 25). Moreover, increasing numbers of studies have indicated that mutations in dihydropteroate synthase genes may be associated with the emergence of TMP/SMX resistant strains (26, 27), especially for patients who taken sulfa as prophylaxis after SOT. Therefore, it is of clinical importance to find a treatment that can both improve the efficacy and reduce the adverse effects on the base of low dose TMP/SMX.

In the TMP/SMX failed PCP cases, pentamidine, atovaquone, dapsone and clindamycin-primaquine can be used as the second-line alternatives, both for HIV-PCP (28) and non-HIV-PCP patients (12, 29). However, no agent has been shown to have better outcomes than TMP/SMX. In severe infections, intravenous pentamidine probably remains the preferred second-line agent after TMP/SMX. However, pentamidine therapy can be complicated by numerous toxicities including pancreatitis, hypo-and hyperglycemia, bone marrow suppression, renal failure, and electrolyte disturbances. Consequently, more and more studies suggest that clindamycin-based alternatives play an increasing role in treating of SOT-PCP, especially for patients who are refractory to TMP/SMX or pentamidine or both (30–33). Clindamycin is a lincosamide agent that inhibits protein synthesis at the chain elongation step by interfering with transpeptidation of the 50S ribosomal subunit. Therefore, the objective of this study is to investigate the safety and efficacy of the preemption clindamycin with low-dose TMP/SMX regimen (CT regimen) for severe PCP after renal transplantation.

## Materials and Methods

### Study Design and Population

We performed a historical-control, retrospective study of PCP patients after renal transplantation, during September 2017 to February 2020. All the patients were admitted with a confirmation of *Pneumocystis* in the blood sample and/or broncho-alveolar lavage (BAL) fluid by “Next-generation” sequencing (NGS) technology (27) and the typical signs listed in the including criteria: (1) Patients were admitted into ICU for distress of respiratory (P/F ratio < 250 mmHg); (2) age >18 years; (3) presented with the symptoms of fever; and (4) tachypnea (respiratory rate >25 breaths/min), and dry cough et al., with diffuse interstitial processes on chest radiograph, but without significant sputum production (34). Pregnant women or terminal stage patients (patients with advanced cancer or severe insufficiency of organ function). All the patients were transferred to ICU immediately after they were admitted. All patients provided informed consent, and this study was approved by the Ethics Committee of Zhongshan Hospital, Fudan University (No. B2019-267R).

**FIGURE 1:**
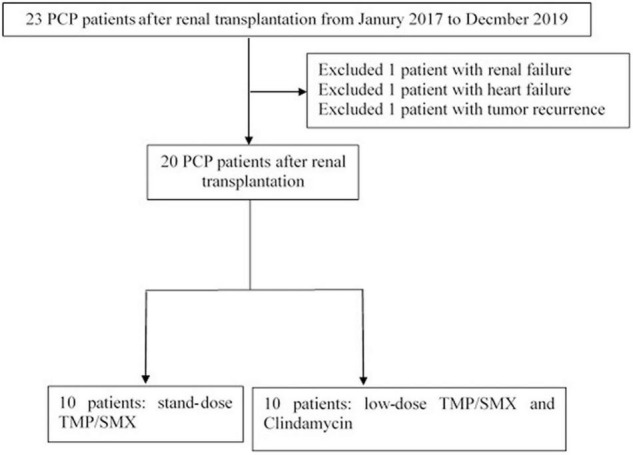
Patient-selection flow chart.

In the first stage of the study (September 1st, 2017 to December 31th, 2018), a total of 12 PCP patients were admitted to our department for hypoxemia. Two patients were excluded, one for renal failure the other one for end stage of carcinoma. Finally, ten patients were included in the study; they were initially treated with the standard dose of TMP/SMX (T group, 15 mg/kg/d TMP). The second stage was from January 1st, 2019 to February 6th, 2020. A total of 11 PCP patients were admitted, while one was excluded for heart failure. Consequently, ten patients were included and treated with the combination of clindamycin and low initial dose of TMP/SMX (CT group, 8 mg/kg/d TMP) (Figure 1). The TMP/SMX-based regimens were initiated from the ICU ward. In both groups, the dosage of TMP/SMX would be modified according to the baseline renal function, white blood cell or platelets count, the clinical effect and the side effect. Clindamycin was initiated at the dosage of was 600 – 900 mg IV or po q6 – 8 h and stopped until the P/F ratio was higher than 300 mmHg and/or the pulmonary infiltration was alleviated.

### Other Interventions Besides Trimethoprim-Sulfamethoxazole and Clindamycin

Upon admission, immunosuppressants were stopped in all patients. According to the protocol from our team, the patients were initially administered with methylprednisolone at 2.0–2.5 mg/kg/day once every 12 h. This dosage was continued until oxygenation improved, followed by gradual tapering (via a 20 mg reduction every 2–3 days) (35). Empirical antibiotic therapy included moxifloxacin, meropenem, and ganciclovir. If fungal infection was suspected, antifungal therapy was initiated. The dosages of all drugs were adjusted based on the allograft function. The heart rate (HR), blood pressure (BP), respiratory rate (RR), and arterial oxygen saturation (SaO_2_) were continuously monitored in all patients. In addition, lung CT scan was performed once every 5–7 days, meanwhile, bedside lung and heart ultrasound was performed twice a day to manifest the pulmonary infiltration, heart function and mediastinal emphysema.

### Protocol for Oxygen Therapy

High-flow Nasal Cannula (HFNC) was considered the first-line treatment if the P/F ratio was under 250 mmHg while using a conventional face mask at a maximum concentration (36). Initially, HFNC was settled with a flow rate of 40–60 L/min and the humidification temperature 31°C. FiO_2_ was modified to maintain a SaO_2_ > 92%. Whenever HFNC could not keep the target SaO_2_, non-invasive ventilation (NIV) or invasive mechanical ventilation (IMV) would be considered.

### Study Outcomes

The primary endpoint was the hospital survival. The secondary endpoints including the length of hospital staying (LOS_*HOS*_), length of ICU staying (LOS_*ICU*_), the time for P/F ratio to 300 mmHg, need for mechanical ventilation (invasive or non-invasive) or extra corporeal membrane oxygenation (ECMO), need for renal replacement therapy (RRT), changes of renal function, pneumomediastinum and superinfection rate.

### Statistical Analysis

Normally distributed data were expressed as mean ± SD and compared with the use of unpaired *t* test. Non-normal data was reported as median (interquartile range) and compared with the use of Mann-Whitney *U* test. Differences between categoric variables, expressed as n (%) were assessed with the use of chi-square or Fisher exact test when necessary. A two-tailed *P* value of < 0.05 was considered to indicate statistical significance. The results were analyzed with the use of SPSS statistical software (version 17.0; SPSS, Chicago, IL, United States).

## Results

### Baseline Characteristics of the Study Population

The mean age was 48.5 and 40.0 years in the T group and the CT group, respectively (*P* = 0.719). There were 8 and 7 male patients in T group and CT group, respectively (*P* = 0.628). The Body Mass Index (BMI) was similar between the T and CT group (21.1 vs. 20.5 kg/m^2^, *P* = 0.481). There was no significant difference in the comorbidities and coinfection between the T and CT group (Table 1). The PSI score between the T and CT group was similar (80 vs. 80, *P* = 0.6). Other clinical characteristics between the CT and the T group on admission, including the APACHE II score (17.5 vs. 14.0, *P* = 0.176), SOFA score (6 vs. 4, *P* = 0.127), the P/F ratio (148.5 vs. 146.0 mmHg, *P* = 0.677), lactate dehydrogenase (LDH, 456.7 vs. 437.2 U/L, *P* = 0.804), C-reactive protein (CRP, 74.9 vs. 67.6 mg/L, *P* = 0.774), procalcitonin (PCT, 0.9 vs. 0.2 ng/mL, *P* = 0.178), total bilirubin (5.9 vs. 9.8 μmol/L, *P* = 0.300), creatinine (223.3 vs. 144.5 μmol/L, *P* = 0.205), platelet (PLT, 219.5 vs. 230.6 × 10^9^/L, *P* = 0.832), hemoglobin (94.4 vs. 103.5 g/dL, *P* = 0.242), leukocyte count (8.1 vs. 10.0 × 10^9^/L, *P* = 0.417), lymphocyte count (0.44 vs. 0.36 × 10^9^/L, *P* = 0.378), CD4^+^/CD8^+^ ratio (1.2 vs. 1.4, *P* = 0.964), globulin (21.8 vs. 19.2 g/L, *P* = 0.239), kalium (4.2 vs. 4.2 mmol/L, *P* = 0.901) and 1,3 – β – D glucan (207.0 vs. 265.0 pg/mL, *P* = 0.887) were all balanced distributed. Nine patients in the CT group and 8 in the T group received HFNC on ICU admission (*P* = 0.556). More patients were administrated with vasopressor in the CT group than in the T group (30 vs. 10% *P* = 0.290).

**TABLE 1:** Baseline characteristics of 20 PCP patients after renal transplantation.

	T group	CT group	*P*
Age (years)	48.5	40.0	0.719^#^
Male (%)	8 (80%)	7 (10%)	0.628^#^
Body Mass Index (kg/m^2^)	21.1	20.5	0.481^#^
Time from transplantation to PCP onset (days)	234.5	313.0	0.161^#^
Comorbidity (n)			
Hypertension (n)	2 (20%)	2 (20%)	1.000*
Smoking (n)	1 (10%)	2 (20%)	0.556*
Diabetes (n)	2 (20%)	2 (20%)	1.000*
Coronary artery disease (n)	2 (20%)	3 (30%)	0.628*
Chronic bronchitis (n)	1 (10%)	2 (20%)	0.556*
Coinfection (n)	4 (40%)	3 (30%)	0.660*
Bacteria (n)	1 (10%)	0 (0%)	0.343*
Fungus (n)	2 (20%)	2 (20%)	1.000*
Virus (n)	1 (10%)	1 (10%)	1.000*
**On admission**			
PSI score	80.0	80.0	0.600^#^
APACHE II score	14.0	17.5	0.161^#^
SOFA score	4.0	6.0	0.127^#^
PaO_2_/FiO_2_ ratio	146.0	148.5	0.176^#^
Vasopressor (%)	1 (10%)	3 (30%)	0.290*
HFNC (%)	8 (80%)	9 (90%)	0.556*
Lactate dehydrogenase (U/L)	437.2	456.7	0.804^#^
C-reactive protein (mg/L)	67.6	74.9	0.774^#^
Procalcitonin (μg/L)	0.2	0.9	0.178^#^
Creatinine (μmol/L)	144.5	223.3	0.205^#^
eGFR (ml/min/1.73 m^2^)	72.0	42.6	0.216^#^
Total Bilirubin (mg/dL)	9.8	5.9	0.300^#^
Platelet count (× 10^9^/L)	230.6	219.5	0.832^#^
Hemoglobin (g/dL)	103.5	94.4	0.242^#^
Leukocyte count (× 10^9^/L)	10.0	8.1	0.417^#^
Lymphocyte count (× 10^9^/L)	0.36	0.44	0.378^#^
CD4^+^/CD8^+^ ratio	1.4	1.2	0.964^#^
Globulin (g/L)	19.2	21.8	0.239^#^
Kalium (mmol/L)	4.2	4.2	0.901^#^
1,3 – β – D glucan (pg/mL)	265.0	207.0	0.887^#^

^#^Mann-Whitney U and Wilcoxon tests; *Fisher exact tests.

### Outcomes

#### Survival and Length of Staying

There were 8 surviving discharge records in the T group, while all the patients survived in the CT group (mortality, 20 vs. 0%, *P* = 0.168). Meanwhile, compared to the T group, CT group had a shorter staying of hospital (26.5 vs. 39.0 days, *P* = 0.011), ICU (12.5 vs. 22.5 days, *P* = 0.008) and less hospital cost (183,694.5 vs. 255,712.0 CNY, *P* = 0.505) (Table 2).

**TABLE 2:** Outcomes of 20 PCP patients after renal transplantation.

	T group	CT group	*P*
Hospital mortality (%)	2 (20%)	0 (0%)	0.168*
Length of hospital staying (days)	39.0	26.5	0.011^#^
Length of ICU staying (days)	22.5	12.5	0.008^#^
HFNC (h)	156.0	168.0	0.616^#^
Mechanical ventilation (%)	4 (40%)	2 (20%)	0.356*
Non-invasive positive-pressure ventilation (NIV, %)	3 (30%)	2 (20%)	0.628*
NIV (h)	17.0	106.8	0.567^#^
Invasive mechanical ventilation (IMV, %)	3 (30%)	0 (0%)	0.211*
IMV (h)	240.0	0.0	0.185^#^
Extra corporeal membrane oxygenation (%)	1 (10%)	0 (0%)	0.343*
Extra corporeal membrane oxygenation (d)	6.0	0.0	0.343^#^
Time for P/F to 300 mmHg (h)	11.0	5.5	0.068^#^
Renal replacement therapy (n)	0 (0%)	1 (10%)	0.343*
Changes of renal function, ^Δ^eGFR (%)	7.15	−19.8	0.400^#^
Renal allograft survival (%)	8 (80%)	9 (90%)	1*
Pneumomediastinum (%)	5 (50%)	0 (0%)	0.008*
Hospital Cost (¥)	255712.0	183694.5	0.505^#^
Average TMP/SMX dosage (mg/d)	146.0	227.4	0.094^#^
Decrement of TMP/SMX dosage	8 (80%)	2 (20%)	0.023*
Transfusion of platelets (%)	4 (40%)	3 (30%)	0.660*
Transfusion of Red blood cell (%)	6 (60%)	3 (30%)	0.196*

^#^Mann-Whitney U and Wilcoxon tests; *Fisher exact tests. ^Δ^eGFR = (eGFR_0_–eGFR_n_)/eGFR_0_.

#### Need for Mechanical Ventilation

During the ICU staying, the length of HFNC application was similar between CT group and T group (168.0 vs. 156.0 h, *P* = 0.616). The non-invasive ventilation (NIV) rate was similar in CT group and in T group (20 vs. 30%, *P* = 0.865), but the invasive ventilation (IMV) rate was relatively lower in CT group than in T group (0 vs. 30%, *P* = 0.211). One patient adopted ECMO in T group, while none of CT group needed. The pneumomediastinum incidence was much higher in the T group than in the CT group (50 vs. 0%, *P* = 0.033) (Table 2).

#### Oxygenation and Pulmonary Infiltration Improvement

**FIGURE 2:**
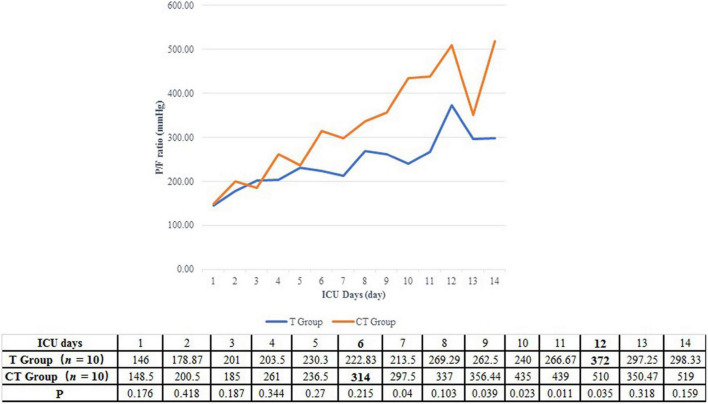
Daily P/F ratio of PCP patients after renal transplantation. For patients in the CT group, the P/F ratio had been elevated to more than 300 mmHg in the 6th ICU day. On the other hand, the P/F ratio of patients from T group could not be higher than 300 mmHg before the 12th ICU day.

**FIGURE 3:**
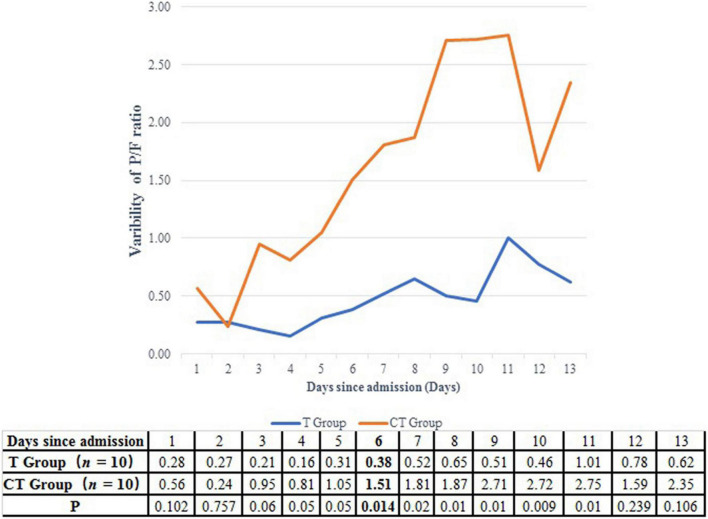
Comparison of the improvement of P/F ratio between CT group and T group. After the 6th ICU day, the improvement of P/F ratio was more significantly in the CT group than in the T group.

A P/F ratio higher than 300 mmHg was fulfilled in a shorter time in the CT group than in the T group (5.5 vs. 11.0 days, *P* = 0.068) (Table 2 and Figure 2). On the other hand, the improvement of P/F ratio [(*P*/*F*_*n*_−*P*/*F*_0_)/*P*/*F*_0_] in the CT group was much more significantly than in the T group after the 6th day after ICU admission (1.51 vs. 0.38, *P* = 0.014) (Figure 3).

Pulmonary tomography scan also indicated that the CT protocol could alleviate the pulmonary infiltration more effectively and quickly than the T protocol, as shown in Supplementary Figure 1.

#### Other Outcomes

The daily fluctuation of the infection markers, hepatic function, renal function and the hematological system from the different therapies were displayed in Supplementary Figure 2. Compared to the T group, CT group was associated with more significant improvement of LDH [(*LDH*_0_−*LDH*_*n*_)/LDH_0_] (0.461 vs. 0.009, *P* = 0.023) (Supplementary Figure 1).

There was no difference in the need for renal replacement therapy (10 vs. 0%, *P* = 0.343), renal allograft survival (90 vs. 80%, *P* = 1), ^Δ^eGFR (−19.8 vs. 7.15%, *P* = 0.400), transfusion of platelets (30 vs. 40%, *P* = 0.660) or red blood cell (30 vs. 60%, *P* = 0.196) between the CT group and T group (Table 2).

#### Adverse Reaction

The adverse reactions of TMP/SMX include rash, anorexia, leucopenia, anemia, thrombocytopenia, hepatic injure, renal injure and hyperkalemia. As for Common Terminology Criteria for Adverse Events 5.0 (CTCAE) ≥ 3 grade adverse reactions, there was no rash, anorexia, renal injure or hyperkalemia in either T group or CT group (Table 3). There was no difference in the occurrence of leucopenia (7/10 vs. 2/10, *P* = 0.07), anemia (6/10 vs. 1/10, *P* = 0.057) or hepatic injure (5/10 vs. 2/10, *P* = 0.350) between the T group than in the CT group. While, more thrombocytopenia (9/10 vs. 3/10, *P* = 0.020) was emerged in the T group than in the CT group. A total of 27 adverse reactions occurred in the T group, while only 8 in the CT group (27/80 vs. 8/80, *P* < 0.001). In the T group, the dosage of TMP/SMX was reduced in 8 patients, while only 2 patients in the CT group received TMP/SMX decrement (*P* = 0.023) (Table 2). The comparison of the daily dose indicated an escalating dose of TMP/SMX in CT group and a decrease dose of TMP/SMX in T group (Supplementary Figure 3).

**TABLE 3:** Present of estimated adverse events from TMP/SMX during the treatment among the groups.

	T Group (*n* = 10)	CT Group (*n* = 10)	*P*
Rash (times)	0	0	1.000
Anorexia (times)	0	0	1.000
Leucopenia (times)	7	2	0.07*
Anemia (times)	6	1	0.057*
Thrombocytopenia	9	3	0.020*
Hepatic injure (times)	5	2	0.350*
Renal injure (times)	0	0	1.000
Hyperkalemia (times)	0	0	1.000
Total AE (times)	27	8	< 0.001^#^

^#^χ^2^ tests; * Fisher exact tests.

## Discussion

In the present study, we found that in comparison to standard dose TMP/SMX, clindamycin combined with low-dose TMP/SMX significantly improved the oxygenation of severe PCP patients after renal transplantation (P/F variability 1.51 vs. 0.38, *P* = 0.014). The CT protocol was also associated with a shorter length of ICU (12.5 vs. 22.5 days, *P* = 0.008) and hospital staying (26.5 vs. 39.0 days, *P* = 0.011) compared with the T group. Meanwhile, the combined drug administration did not increase the occurrence rates of hepatic or renal toxicity, but rather reduced the severe adverse reactions of TMP/SMX (27/80 vs. 8/80, *P* < 0.001) and eventually improve the compliance.

*Pneumocystis* pneumonia patients, especially the non-HIV-PCP patients were usually associated with poor outcome (17, 24, 28). In the present study, the hospital mortality of severe PCP in standard dose TMP/SMX was 20% which was similar to the previous report (24). In comparison, all the patients in CT group were discharged. In the current study, the LOS_*ICU*_ of PCP patients of the T group was 22.5 days, which was similar to the previous study (37). When the combination protocol was applied, the LOS_*ICU*_ significantly reduced from 22.5 to 12.5 days (*P* = 0.008) and LOS_*HOS*_ reduced from 39.0 to 26.5 days (*P* = 0.011). Moreover, the CT protocol could alleviate the pulmonary infiltration more effectively and quickly than the T protocol. Therefore, we proposed that the combination of clindamycin and low-dose TMP/SMX could improve the outcome of severe PCP after renal transplantation.

The clinical benefit of CT protocol may be due to the following mechanisms. First, CT protocol can improve patients’ oxygenation in a better and quicker way. We found that although the initial P/F ratio was similar between the CT group and the T group, patients in the CT group spent only 5.5 days to achieve the P/F ratio > 300 mmHg, while patients in the T group need 11 days. Moreover, after the 6th ICU day, patients in the CT group displayed a more significant P/F ratio improvement then patients in the T group (1.51 vs. 0.38, *P* = 0.014). In line with the more effective oxygenation improvement, the ratio of IMV was accordingly lower in the CT group than in the T group (30 vs. 0%). Mechanical ventilation had confirmed as the independent predictor of increased mortality for HIV-PCP patients (38, 39).

Second, pneumothorax or pneumomediastinum is also associated with worse outcomes of PCP, with one study citing an increase in mortality up to 50% (40). We found that half of the T group got pneumomediastinum while no one of the CT group got it. We propose that the higher IMV rate in the T group might lead to higher incidence of spontaneous pneumothorax or pneumomediastinum because IMV could increase the pressure of the alveoli that can result in alveolar rupture (41). Pneumothorax or pneumomediastinum may be also a result of severe inflammation and fibrosis from PCP (42). We found that CT group was associated with significant improvement in LDH (*P* = 0.023, Supplementary Figure 1). Several studies have reported that elevated serum LDH levels were associated with the severity of several pulmonary disorders (43–45). Our team had previously demonstrated that elevated LDH was associated with 90-day mortality in renal transplant recipients with severe CAP (46). Elevated serum LDH levels were mainly due to the impaired pulmonary parenchymal cells or local inflammatory cells, such as alveolar macrophages and polymorphonuclear neutrophils (47). Therefore, the improvement in pulmonary injury and reduced the pneumothorax or pneumomediastinum probability were referred to a significant reduction of LDH by CT protocol.

Although it had been known that TMP/SMX treated PCP by interfering folate metabolism in pneumocystis (48), there are few studies about how did clindamycin treat PCP. Up to now, clindamycin was known to have the following therapeutic mechanisms: (1) Inhibit the protein synthesis in a parasite-specific organelle (the apicoplast) (49), which was related to the mitochondrial function and the lifecycle; (2) Reduce the protein and nucleic acid synthesis in *Plasmodium falciparum* (50). We proposed that the different effects from TMP/SMX and clindamycin could create a 1+1 > 2 effect in PCP treatment. We are also preparing to explore to the underlying mechanisms of the CT protocol.

There was a lower initial TMP/SMX dose in the CT group than in the T group (Supplementary Figure 3), while it was finally found that there was a relatively higher mean daily dose of TMP/SMX in CT group than in the T group (227.4 vs. 146.0 mg TMP, *P* = 0.094). There existed the following reasons. First of all, in the current study, patients were more fragile to the standard or high dose of TMP/SMX for their poor eGFR from the renal allograft. Second, clindamycin could facilitate patients recover from PCP together with the recovery of renal function through the pathway different from TMP/SMX. Therefore, we proposed that for severe PCP after renal transplantation, the higher initial dose of TMP/SMX was an important risk factor for severe adverse reactions of TMP/SMX (27/80 vs. 8/80, *P* < 0.001) and clindamycin could help to create a suitable state when patients could tolerant an escalating dose of TMP/SMX. In addition, our results indicated that for severe PCP after renal transplantation, the higher total TMP/SMX dose was more important than the higher initial dose for a good outcome.

This study is the first one that provide preliminary evidence to support the combination of clindamycin and low-dose TMP/SMX for severe PCP patients after renal transplantation especially when they were intolerant to the standard dose of TMP/SMX for the poor renal function. However, several limitations exist. First, this is not a random design, but a single-centered retrospective observation study. Second, the cohort volume is small. Finally, the underlying mechanisms for CT protocol to treat PCP is not explored in the current study. We are looking forward to carry out a multicenter RCT trial and the *in vivo* or *in vitro* study to reinforce the current results.

## Conclusion

The current study proposed that clindamycin combined with low-dose TMP/SMX was more effective and safer than single use of TMP/SMX for severe PCP patients after renal transplantation.

## Data Availability Statement

The original contributions presented in the study are included in the article/Supplementary Material, further inquiries can be directed to the corresponding author.

## Ethics Statement

The studies involving human participants were reviewed and approved by the Ethics Committee of Zhongshan Hospital, Fudan University. The patients/participants provided their written informed consent to participate in this study.

## Author Contributions

M-JJ, Z-YG, D-LH, W-JL, and Y-JL involved the study design and manuscript preparation. Z-YG, D-LH, Y-ML, and H-YH involved in the data collection and data analysis. M-JJ and CY involved in the statistical design. MX, R-MR, D-MZ, and ZL involved in the manuscript preparation. All authors read and approved the final manuscript.

## Conflict of Interest

The authors declare that the research was conducted in the absence of any commercial or financial relationships that could be construed as a potential conflict of interest.

## Publisher’s Note

All claims expressed in this article are solely those of the authors and do not necessarily represent those of their affiliated organizations, or those of the publisher, the editors and the reviewers. Any product that may be evaluated in this article, or claim that may be made by its manufacturer, is not guaranteed or endorsed by the publisher.
