# Supplementary material for: Preliminary Study on the Combination Effect of Clindamycin and Low Dose Trimethoprim-Sulfamethoxazole on Severe Pneumocystis Pneumonia After Renal Transplantation
Source: Front Med (Lausanne). 2022 May 6;9:827850. doi: 10.3389/fmed.2022.827850 (PMC9120531; doi:10.3389/fmed.2022.827850)
Supplement: Supplementary file 1 [file Data_Sheet_1.docx]

**Figure S1 Change of pulmonary infiltration in the pulmonary tomography.**





The pulmonary infiltration was significantly improvement in one typical patient from CT group (A, ICU admission; B, 7^th^ ICU day). On the other hand, in one typical patient from T group the pulmonary infiltration was not significantly improvement during the first week in ICU (C, ICU admission; D, 7^th^ ICU day).

**Figure S2 Daily comparisons of the infection markers, hepatic function, renal function and the hematological system between CT group and T group**

**
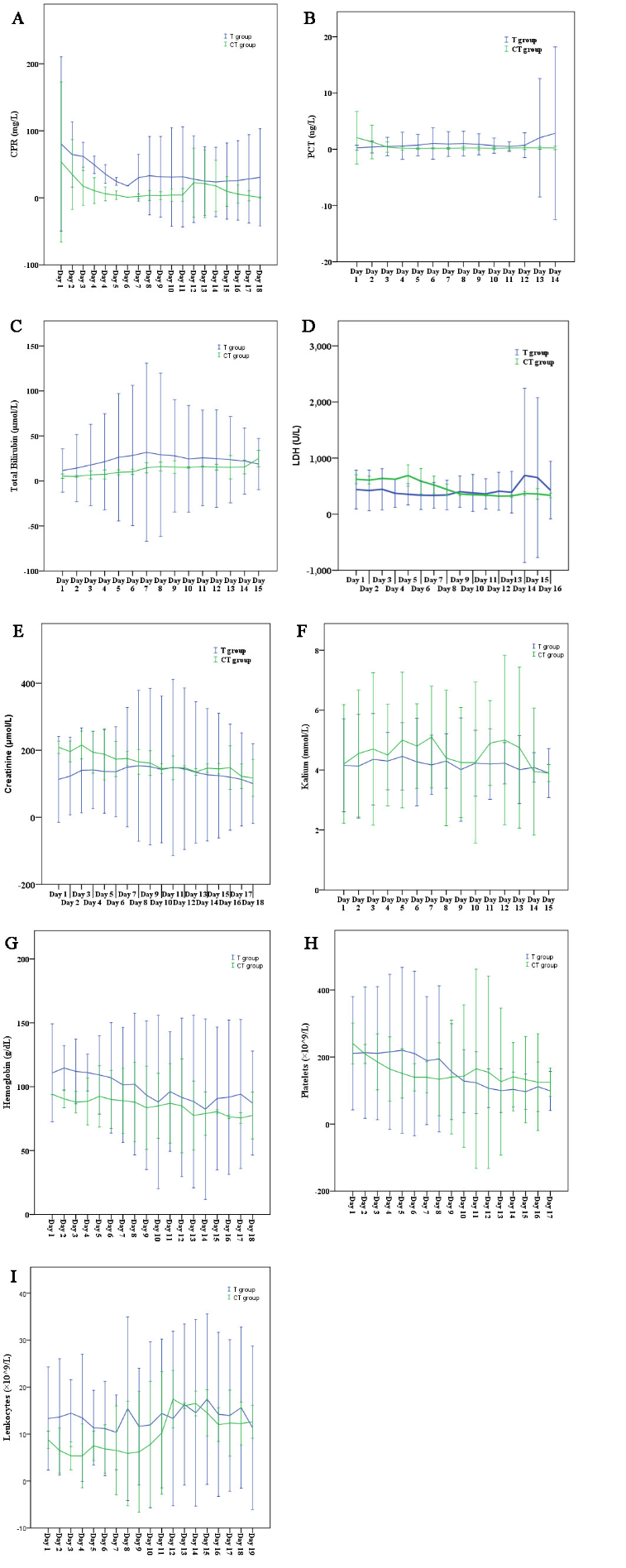
**

There was only the LDH level was significantly improved in the CT group when compared to the T group. (D)

Figure S3. The comparison of the daily dose of TMP/SMX between CT and T group.


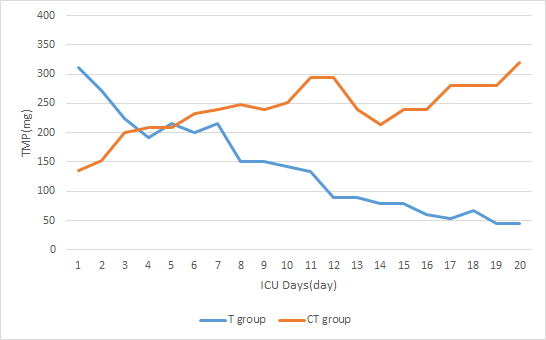


The comparison of the daily dose indicated an escalating dose of TMP/SMX in CT group and a decrease dose of TMP/SMX in T group
